# Critical Role of Gut Microbiota and Epigenetic Factors in the Pathogenesis of Behçet’s Disease

**DOI:** 10.3389/fcell.2021.719235

**Published:** 2021-10-05

**Authors:** Xiaomin Ma, Xin Wang, Guangbing Zheng, Guiqin Tan, Fangyu Zhou, Wenwen Wei, Dan Tian, Hongsong Yu

**Affiliations:** School of Basic Medical Sciences, Special Key Laboratory of Ocular Diseases of Guizhou Province, Zunyi Medical University, Guizhou, China

**Keywords:** Behçet’s disease, gut microbiota, epigenetics, DNA methylation, histone modification, microRNA

## Abstract

Behçet’s disease (BD) is a chronic refractory multisystem autoinflammatory disease, characterized by typical clinical features of non-specific vasculitis, oral and genital ulcers, uveitis, as well as skin lesions. The exact etiopathogenesis of BD remains unknown, existing studies have indicated that genetics and environmental factors contribute to the increased development of BD. Recently, several studies have shown that external environmental factors can affect the process of epigenetic modification, and abnormalities of epigenetic factors have been confirmed to be involved in the occurrence of BD. At the same time, abnormalities of gut microbiota (GM) in the body, have also been confirmed to participate in the pathogenesis of BD by regulating the balance of Th17/Tregs. This article reviews the pathogenesis of BD and summarizes numerous clinical studies, focusing on the mechanism of GM and epigenetic factors impacting on BD, and providing new ideas for further elucidating the pathogenesis of BD.

## Introduction

Behçet’s disease (BD) is a recurrent, chronic, multisystem autoinflammatory disease, characterized by recurrent stomatitis, uveitis, genital ulcer, oral ulcer, and skin damages (Alipour et al., 2017; Ortiz-Fernández and Sawalha, 2021). There are obvious regional and gender differences in the incidence of the disease. BD is most common along the ancient Silk Road stretching from China to the Middle East, such as the Mediterranean, Middle East, and far East (Cho et al., 2012; Chen et al., 2017). A meta-analysis showed that the highest prevalence rate (expressed as cases/100,000 inhabitants) is 119.8 for Turkey, 31.8 for the Middle East, 4.5 for Asia, and 3.3 for Europe (Maldini et al., 2018). In the same Asian region, the incidence rate in South Korea is 1.51/100000, which is common in women (Jun et al., 2020). While it is about 14/100000 in China, the prevalence rate is significantly higher in males than in females (Yang et al., 2021). These differences may be due to the inconsistency of other factors such as research methods. Although accumulating evidence has shown that many genetic factors, such as *HLA-B51*, *IL1A-IL1B*, *CEBPB-PTPN1*, *IRF8*, *ADO-EGR2*, *RIPK2*, and *LACC1*, are involved in the susceptibility of BD, the exact etiology of BD remains unclear (Takeuchi et al., 2017).

A number of studies have shown that the differentiation of helper T cells and the expression of corresponding inflammatory cytokines are abnormal in patients with BD. Recently, several studies have shown that the number of regulatory T cells (Tregs) decreases in patients with BD, and the corresponding main anti-inflammatory cytokine interleukin 10 (IL-10) and TGF-β are also significantly decreased. This abnormality may lead to damage of the immunosuppressive state, which leads to the autoimmune environment in the pathogenesis of BD. In addition, the proportion of T helper 17 (Th17) cells and the level of IL-17 and IL-23 in patients with active BD are significantly increased (Hamzaoui et al., 2011; Zhu et al., 2017; Vural et al., 2021). Studies have found that serum IL-26 levels are significantly increased in patients with active BD (Lopalco et al., 2017). A study proposed that IL-26 can promote the production of Th17 (IL-17A, IL-23) and inhibit the production of Treg (IL-10, TGF-β) by stimulating CD4 + T cells and monocytes (Kaabachi et al., 2017). The expression of proinflammatory cytokines IL-6, IL-1 β, and tumor necrosis factor alpha (TNF-α) in dendritic cells (DCs) of active BD patients was significantly higher than that of healthy controls (Liang et al., 2021). Even in the peripheral circulation, the level of IL-9 mRNA in BD patients was higher than that of healthy controls, which was positively correlated with the level of IL-17 (Kaabachi et al., 2019). The proportion of Th17/Tregs in BD patients is much higher than that in healthy controls (Acosta-Rodriguez et al., 2007; Wilson et al., 2007). Therefore, the pathogenesis of BD may be due to immune tolerance deficiency caused by the decrease of Tregs, while the increase of Th17 cells promotes inflammation. Recent studies have confirmed that changes of intestinal microorganisms participate in the occurrence and development of BD by regulating Tregs, while external environmental factors also regulate the expression of Th17/Tregs ratio through epigenetic processes and are closely related to BD. Therefore, this article reviews the role of the association of intestinal microbiota and epigenetic factors with the etiology of BD.

## The Gut Microbiota and Behçet’s Disease

Gut microbiota (GM), which is considered a metabolic organ, are involved in regulating host metabolism and is a vital factor in maintaining human health and balance in body. Short-chain fatty acids (SCFAs) are the downstream mediators of GM anti-inflammatory activity, which can regulate the mucosal immune system (Consolandi et al., 2015; Shimizu et al., 2019). The synergistic effects of these metabolites are the basis for maintaining immune homeostasis and host immune system function (Arpaia et al., 2013). The imbalance of GM may lead to pro-inflammatory responses, and changes in GM composition regulate the progression of many human inflammatory autoimmune diseases, such as systemic lupus erythematosus (He et al., 2020), psoriasis (Zhang et al., 2021), and rheumatoid arthritis (Brandl et al., 2021). Abnormal activities of Th1 cells, Th17 cells, and Tregs have been observed in patients with BD, and changes in the composition and metabolism of GM play a role in immune abnormalities in BD through the imbalance between Th17 cells and Tregs (Round and Mazmanian, 2009; Ye et al., 2018; Oezguen et al., 2019; van der Houwen et al., 2020; Yan et al., 2020). Therefore, the changes in GM are closely related to BD.

A research team from Italy analyzed the total bacterial DNA in the feces of 22 BD patients and 16 matched healthy controls. It was reported for the first time that a peculiar dysbiosis of the GM is present in BD patients, with a significant decrease in butyric acid production. *Roseburia* and *Subdoligranulum* in the GM were also significantly depleted. Butyric acid is a beneficial metabolite of SCFAs, which can protect the integrity of the intestinal epithelial barrier and affect immune regulation and mucosal immune response by inducing Tregs differentiation. Intestinal butyrate can also inhibit local pro-inflammatory cytokines. The reduced level of butyric acid leads to intestinal epithelial barrier dysfunction, promotes the expression of various inflammatory components, and reduces the level of Tregs which may promote an abnormal immune response (Consolandi et al., 2015). A study of the Japanese population found that the relative abundance of *Clostridia* in patients with BD was decreased. *Clostridia*, including SCFA-producing bacteria. The decrease in its abundance led to the reduced of SCFAs concentration, resulting in dysregulation of immune function in patients with BD. In addition, it was also found that the species of megalomonas and Vibrio butyricum producing SCFAs were also decreased, which may lead to the depletion of SCFAs in the intestine. The data show that the abnormality of GM in BD changes the synthesis of nucleic acids and fatty acids, and these changes in composition and function may be accompanied by adverse molecular exchanges between intestinal immunoreactive cells and intestinal microorganisms, which may be related to immune abnormalities in patients with BD (Shimizu et al., 2019). A study in a Dutch cohort found that in patients with BD, the abundance of *Barnesiellaceae* and *Lachnospira* was decreased. *Barnesiellaceae* may exert protective anti-inflammatory effects by reducing the level of TNF-α, one of the key and targeted cytokines of BD, and the decrease in butyric acid production may be regulated by reducing the abundance of *Lachnospira*, thereby affecting T-cell differentiation and causing inflammation in BD. GM participate in the occurrence and development of BD mainly by Tregs and affecting the balance of Th17/Tregs, but there are also some bacteria that play a role through other mechanisms (van der Houwen et al., 2020).

A report proposed that the relative abundance of *Eggerthella lenta*, *Acidaminococcus* species, *Lactobacillus mucosae*, *Bifidobacterium bifidum*, *Lactobacillus*, *Streptococcus*, and *saliva Lactobacillus* in the feces of patients with BD was significantly increased (Shimizu et al., 2019). In another study, it was found that at the genus level, *Eggerthella* was significantly increased in BD patients, whereas the relative abundance of *Megamonas* and *Prevotella* was significantly decreased. The role of Bacilli in inducing systemic inflammation was consistent and *Lactobacillus* plays a relatively large role in the BD microbiota (Shimizu et al., 2016). The researchers used IgA sequencing analysis to reveal that the species of *Bifidobacterium*, *Dorea*, and *Ruminococcus* coated with IgA increased, indicating that these microorganisms drive specific immunostimulatory responses, which may be pathogenic symbiotic bacteria in BD, reflecting the retention of anti-inflammatory species and neutralization of pathogenic symbiotic bacteria in BD. IgA coating of *Bifidobacterium* and *Brominated rumencocci* induced in BD may effectively retain bacteria in intestinal mucosa and promote a sustainable dynamic balance by inhibiting pro-inflammatory signals in the host (van der Houwen et al., 2020). A research team from China analyzed the fecal and saliva samples of active BD patients and healthy controls, and found that several opportunistic pathogens were enriched in BD patients, while methanogens and butyrate-producing bacteria (BPB) were enriched in healthy controls. The overgrowth of conditional pathogenic bacteria may disrupt the balance of GM, leading to the decrease of BPB and methanogens. These abnormalities may lead to damage of the intestinal epithelial barrier, and promote the entry of effector molecules or MAMP/PAMP (PGN/LPS) into intestinal epithelium. At the same time, this process induces the overexpression of the corresponding pattern recognition receptor (TLR2/TLR4), which leads to a series of inflammatory reactions, including systemic vasculitis of BD (Ye et al., 2018). In another study, hypomethylated *TLR4* promoter and increased *TLR4* expression were found in BD patients, which suggests that there may be a common pathogenic pathway between GM and epigenetic factors (Kolahi et al., 2020). To determine whether GM play a role in the development of BD, researchers transplanted mixed fecal samples from patients with active BD into mice with experimental autoimmune uveitis (EAU) and observed increased intraocular inflammation; a large amount of inflammatory cell infiltration throughout the retina, choroid, and vitreous cavity; and increased production of inflammatory cytokines including IL-17 and interferon gamma (IFN-γ) (Ye et al., 2018). These findings further confirmed that imbalance of the GM may indeed be involved in the occurrence and development of BD. From the above, it can be seen that the decrease in the abundance of SCFAs play a particularly important role in the special flora imbalance of BD.

Recent studies have reported that fecal microbiome transplantation (FMT) can promote the increase of butyric acid-producing bacteria, so as to achieve the effect of treatment. The researchers transplanted human feces with normal glucose tolerance into type 2 diabetic mice. After FMT treatment, the level of SCFAs in diabetic mice increased, and the level of butyric acid increased significantly after 10 weeks of treatment. It is speculated that the mechanism may be that it can increase the diversity of GM in diabetic mice, affect different kinds of microorganisms in the intestinal tract of mice, and increase the number of bacteria that produce SCFAs, thus increasing the content of SCFAs in the feces of diabetic mice, and regulating dysfunctional glucose and lipid metabolism (Han et al., 2021). Therefore, the in-depth study of GM and FMT in patients with BD may provide new methods and ideas for the treatment of the disease. In addition, a study has found that patients with BD have special flora disorders, and the comparative analysis of the GM in BD, familial Mediterranean fever and Crohn’s disease, which have the similar innate and autoinflammatory features in the pathogenesis, showed that *Succinivibrio* and *Mitsuokella* were “BD specific genera” (Tecer et al., 2020). Therefore, the study of GM may also provide a new direction for the differential diagnosis of BD from other inflammatory diseases.

Gut microbiota plays a key role in the immune response mainly by regulating the differentiation of T cells. Changes in the number and types of GM lead to immune abnormalities and diseases. The decrease in relative abundance of *Clostridium* can reduce the production of SCFAs, such as butyric acid, thus reducing the differentiation of Tregs, disrupting the balance of Th17/Tregs, and playing a role in the onset and development of BD. In addition, GM also participate in the BD process by driving specific immunostimulatory responses. The related research of GM is currently a hot spot, and further research in this area will promote our understanding of the pathogenesis of BD.

## Epigenetics and Behçet’s Disease

Epigenetics refers to the heritable changes of gene expression without changing the DNA sequence, that is, phenotypic changes without changing genotypes (Alipour et al., 2017). It plays an important role in controlling gene expression and regulating cell development, differentiation, and activity (Allis and Jenuwein, 2016). It can not only maintain specific cell lines stably but also dynamically respond to developmental and environmental signals (Muhammad et al., 2019). Epigenetics are collection of mechanisms by which environmental stressors affect gene expression rather than potential genetic sequences (Barrere-Cain and Allard, 2020). Environmental factors and genetic polymorphisms of inflammatory cytokines induce susceptibility to the disease. Some studies have found that epigenetics is a bridge between environment and heredity. It is an important link among the genome, environment and disease, that is, genetic factors control individual susceptibility to disease, while epigenetics ultimately determine the occurrence and phenotype of disease through environmental factors (Hanson and Gluckman, 2008). Epigenetic processes including DNA methylation, histone modification, non-coding RNA (ncRNA), and especially microRNA (miRNA) are thought to be associated with the pathogenesis of BD (Leccese and Alpsoy, 2019). Some genetic or epigenetic factors combined with imbalances in immune regulation lead to the development of BD (Hou et al., 2020). Changes in the methylation level of IRS elements in diffuse repeat sequences, histone modification, and miRNA regulation-mediated gene silencing play a role in the control of immune and inflammatory responses and are related to the pathogenesis of autoimmune diseases, but the specific mechanisms are unknown (Alipour et al., 2017; Leccese and Alpsoy, 2019).

Epigenetics are reversible, and the study of cellular and molecular epigenetic changes associated with BD will provide novel targets for treatment and may address ethnic differences in genetic research verification. Therefore, this paper reviews the role of epigenetic abnormalities in the occurrence and development of BD, such as DNA methylation, histone modification, and ncRNA.

### DNA Methylation and Behçet’s Disease

DNA methylation is the main epigenetic modification of a stable autoimmune disease. In mammals, using S-adenosylmethionine as a methyl donor, methyl is added to the fifth carbon of the cytosine base under the catalysis of DNA methyltransferase, mainly in CpG dinucleotides (Ehrlich et al., 1982; Angeloni and Bogdanovic, 2019). It is generally believed that genetic and environmental factors can change the state of DNA methylation, and the loss of methylation is related to several cancers, autoimmune diseases and inflammation. Animal experiments have shown that DNA methylation inhibitors, such as 5-azacytidine, can control autoimmune diseases, so the study of methylation will provide a better understanding of the disease as well as ideas for treatment (Alipour et al., 2017; Muhammad et al., 2019). In recent years, many studies have confirmed that aberrant DNA methylation is involved in the pathogenesis of BD.

Hyperactivity of neutrophils is an important factor in the immune disorder of BD. Increased levels of cytokines produced by T cells, such as IL-2, IL-12, IL-18, and TNF-α, may lead to the activation of endothelial cells and neutrophils, so the inflammatory response of BD can be attributed to the excessive production of pro-inflammatory cytokines and a decrease in anti-inflammatory factors (Aziz et al., 2020). At present, research on cytokine gene expression is mainly focused on the DNA methylation status of the CpG site (Alipour et al., 2018). A study found that the expression of IL-6 in the peripheral blood mononuclear cells (PBMCs) was significantly higher in patients than in the healthy control group, while the relative promoter methylation level of IL-6 mRNA was significantly decreased. IL-6 is an important cytokine in the pathogenesis of BD and plays a key role in the differentiation of CD4^+^ T cells into Th17 cells. It may affect the expression of effective genes by changing the DNA methylation pattern, thus stimulating the immune response, lowering the methylation level of the IL-6 promoter, and increasing the prevalence of BD (Alipour et al., 2020). Therefore, IL-6 can be used as a molecular marker for the diagnosis of BD, and this difference can be used for the early diagnosis and rapid treatment of disease. Meanwhile, hypermethylation of IL-10 and low levels of gene expression in the PBMCs of BD individuals and reduced serum levels have been confirmed, suggesting that the abnormality of DNA methylation may lead to inactivation of the IL-10 gene in patients with BD. IL-10 can inhibit the proliferation of CD4^+^ T cell clones and reduce inflammation, thus controlling the immune response. At the same time, the study found that the level of methylation was significantly different among different age groups and disease severity. The level of IL-10 methylation is significantly increased in people over 45 years old and is more obvious in patients with severe versus non-severe BD. Demethylation therapy can regulate the expression of IL-10 and control the progression of BD disease (Alipour et al., 2018). And the methylation level of TNF-α in patients with BD was significantly lower than that in normal controls, and the methylation level was significantly different among the subgroups of age, ocular involvement and severe ocular involvement. The more serious the ocular involvement, the lower methylation level of TNF-α. IL-10 and TNF-α are mainly secreted by Tregs, so abnormalities in their methylation may also cause immune disorder by regulating the level of Th17/Tregs, leading to the occurrence of BD (Aziz et al., 2020).

A two-stage study on venous blood samples from 100 Chinese Han patients with active BD and 100 normal controls was found that five differentially methylated CpG loci showed significant hypomethylation of four different genes. Pyrophosphate sequencing verified that cg03546163, cg25114611, cg23261343, and cg142905764 CpG sites with abnormal methylation status can be used as diagnostic markers of BD, in which the hypomethylation of *FKBP5* promoter is the most significant, while the expression of *FKBP5* gene is high. And studies have shown that the hypomethylation of *FLJ43663* and *NFIL3* are associated with BD in Chinese Han population for the first time, but the exact effects of these genes need to be confirmed by further study (Yu et al., 2019). A study found that the methylation level of the *Bax* gene in the PBMCs of patients with BD was significantly higher than that in healthy subjects, while the average value of gene expression decreased. *Bax* is a pro-apoptotic gene that promotes physiological cell death, and the expression level is decreased in patients with severe disease. In addition, the regulation of decreased gene expression caused by methylation occurs under 45 years, but the specific mechanism is not clear (Asadi et al., 2020). In another study, researchers detected suppressor cytokine signaling 1 (*SOCS1*) methylation level in the PBMCs of 50 BD patients and 60 healthy subjects. *SOCS1* methylation level was higher in the patients than in the controls, while the change in *SOCS1* gene expression was less than that in the normal control group. *SOCS1* hypermethylation can activate the Janus kinase/signal transducer and activator of transcription (JAK/STAT) signaling pathway and directly influences the effects of IL-6 and other cytokines on cell function. *SOCS1* also inhibits the production of IFN-γ and IL-17A driven by STAT1 and STAT3 by maintaining the expression of forkhead box protein 3 (FoxP3), which plays an important role in the function and accuracy of Tregs. *SOCS1* plays a complex role in regulating IL-4, IL-12, and IL-15. Therefore, aberrant methylation of *SOCS1* may be involved in the occurrence of BD through these regulatory actions (Abdi et al., 2018).

Changes in innate immune function play an important role in initiating the BD inflammatory response. TLR is the main regulator of the innate immune system. The epigenetic mechanism, especially DNA methylation, controls TLR-related immunity (Muhammad et al., 2019). The expression of *TLR4* mRNA in the PBMCs of patients with active BD was significantly higher than that of the control group, and the methylation rate of the *TLR4* gene promoter in the active and inactive BD groups was significantly lower than that in the control group (Kolahi et al., 2020). Studies have shown that *TLR4* can promote the differentiation of initial CD4^+^T cells into Th17 cells, so the hypomethylation of *TLR4* gene may participate in the pathogenesis of BD by increasing the expression of *TLR4* (Bartlett et al., 2018). In addition, a study found that the methylation levels of CG-7.8.9 unit of *GATA_3*, CG-2 site of *IL-4*, CG-2.3.4.5, and CG-10.11 sites of *TGF-*β in CD4^+^T cells of active BD patients were significantly increased. As an important transcription factor that regulating the differentiation of Th2 cells and the expression of Th2 cytokines, the ablation of *GATA_3* leads to an increase in DNA methylation at the IL-4 gene site and reduced Th2 cytokine production. *TGF-*β is an important multipotent cytokine, which can induce inflammation by promoting the development of Th17 cells and inhibit the immune response by promoting the development of Tregs. Aberrant methylation of *GATA_3*, *IL-4*, and *TGF-*β promoters may participate in BD by regulating T-cell differentiation, but whether it may become a potential biomarker for the disease remains to be further studied (Zhu et al., 2017). Recently, a genome-wide DNA methylation study demonstrated that the reversal of DNA methylation changes in some cytoskeleton-related genes is related to the remission of the BD. Studies have confirmed that there is a dynamic relationship between genetic susceptibility and environmental inducements. These environmental factors can transmit their destructive effects by affecting intracellular epigenetic events (Renauer et al., 2016).

The epigenetic remodeling of cytoskeleton genes underlying the pathogenesis and treatment response of BD provides new and specific molecules, which can be used as therapeutic targets and may develop into biomarkers of disease.

### Histone Modification and Behçet’s Disease

Histone modification refers to the process of histone methylation, acetylation, phosphorylation, adenylation, ubiquitin, or small ubiquitin-like modifier (SUMO) modification under the action of related enzymes, and then regulates the dynamic chromatin structure and gene expression (Bannister and Kouzarides, 2011; Alipour et al., 2017). The most frequently studied histone modification is the modification of the N-terminal H3 (H3K) lysine residue of the histone, which acetylates to neutralize the positive charge, thus reducing the affinity between histone and DNA and promoting transcription in most cases. Histone methylation generally occurs at the lysine and arginine residues of histones H3 and H4, which can be monomethylated, dimethylated (lysine and arginine), or trimethylated (lysine). Misregulation of the methylation process may lead to some diseases, such as cancer and autoimmune diseases (Hanson and Gluckman, 2008; Alipour et al., 2017; Silva et al., 2020). Histone modification plays an important role in a variety of autoimmune diseases, including BD.

Sirtuin 1 (Sirt1) is a histone deacetylase dependent on NAD^+^ coenzyme. It can inhibit T-cell proliferation and proinflammatory cytokine production by modifying histone deacetylation, regulating gene expression, and then regulating cell function and inflammation (Chadha et al., 2019). Researchers treated Sirt1 activator *in vitro* with mouse pLN cells and PBMCs from normal people in patients with BD ophthalmopathy. The proportion of Tregs in the retina decreased after Sirt1 activation. Inhibition or deletion of Sirt1 allowed accumulated acetylated FoxP3 to be protected from proteasome degradation, which enhanced the inhibition of Tregs *in vitro* and *in vivo* (Gardner et al., 2013). SIRT1-deficient mice showed maladjusted peripheral T-cell tolerance (Beier et al., 2011). Related studies have shown that cytokines, such as IL-6 and IL-17, play an important role in the pathogenesis of BD, and that IL-6 particularly plays a key role in promoting Th17 cells to produce IL-17, TNF-α, and IL-6. The ability of Sirt1 activation to inhibit IL-6 production in EAU indicates that Sirt1 activation has a potential limiting effect on Th17 cells and emphasizes that Sirt1 activation may directly or indirectly affect leukocyte recruitment and migration (Gardner et al., 2013). Resveratrol, a small molecule agonist of Sirt activity, can enhance chromatin-related Sirt1 protein in the CIAP-2 promoter region and correct local tissue inflammation and neurotoxicity by inhibiting the activation of microglia (Chen et al., 2005). By feeding resveratrol to endotoxin-induced uveitis mice with lipopolysaccharide-induced uveitis, a study team found that resveratrol could significantly enhance the expression of *Sirt1* gene in retinal pigment epithelial cells and choroid, and inhibit the occurrence of ocular inflammation. This effect was related to the loss of NF-κB regulated gene expression and the sensitivity of cells to TNF-α induced apoptosis (Kubota et al., 2009). The activation of SIRT1 promotes TNF-α induced apoptosis and inhibits NF-κB transcription by inhibiting the transactivation potential of RelA/p65 protein (Yeung et al., 2004; Guenane et al., 2006). Therefore, the regulation of histone acetylation, especially the activation of SIRT1, is a feasible target for the generation of new anti-inflammatory therapies, and the future targeted activation of SIRT1 is expected to become a potential treatment for non-infectious diseases such as BD-related uveitis.

In addition, ubiquitination reactions are involved in the regulation of receptor tyrosine kinase signal and may play important roles in the TNF-α, IL-1β, and TCR-mediated NF-κB activation pathway. Meanwhile, NF-κB regulates apoptosis-related factors and increases T-cell resistance to apoptosis (Hou et al., 2012). There is a strong Th1 cell immune response in active BD. IL-12 can prevent spontaneous and CD95-induced cell death, while the production of IL-12 is directly regulated by NF-κB (Todaro et al., 2005). Therefore, the ubiquitin-related pathway may play a protective role in the occurrence and development of BD, and ubiquitin deficiency may be involved in the pathogenesis of BD. The ubiquitin-related domain coding gene *UBAC2* is associated with susceptibility of the Chinese Han people to BD (Nakamura et al., 2019). SUMO4 participates in autoimmunity and inflammation by regulating NF-κB and activating heat shock transcription factors, resulting in the decreased transcription of proinflammatory cytokines (Hou et al., 2008), some studies have shown that SUMO4 +438 C and −847 G alleles seem to be associated with susceptibility to BD, and their gene polymorphisms may be involved in the development of skin lesions, vascular BD, as well as the severity of the disease (Kamoun et al., 2010). However, how the two participate in the occurrence and development of BD has not been clarified.

In summary, histone modification is involved in the occurrence and development of BD. Sirt1 inhibits the differentiation of Tregs and disrupts immune tolerance by inducing histone acetylation, which leads to the occurrence of BD. In addition, its methylation and ubiquitin abnormalities are also related to BD, although this needs to be confirmed. The specific mechanism of its participation in the occurrence of BD also needs to be further studied.

### Non-coding RNA and Behçet’s Disease

Non-coding RNA refers to RNA, which does not encode proteins. The common characteristic is that it can be transcribed from the genome but not translated into proteins, and can perform their biological functions at the RNA level. NcRNA is an important regulator of the inflammatory immune response, and its genetic variation may affect this biological function. NcRNA involved in epigenetic processes varies according to sequence length. It can be divided into short ncRNA (<30 nucleotides) and long ncRNA (lncRNA) (>200 nucleotides) (Hanson and Gluckman, 2008; Alipour et al., 2017; Hou et al., 2020). NcRNA may be used for disease diagnosis and/or treatment in the future (Akbaba et al., 2020).

#### Long Non-coding RNA and Behçet’s Disease

Long ncRNA is a ncRNA, with a length of more than 200 base pairs that regulates the transcriptional activity of specific genes and even chromosomal regions (Akbaba et al., 2020). It plays a key role in different biological processes, including chromatin remodeling, transcription and epigenetic regulation, as well as the development of various immune cells.

Many studies have shown that there are several specific expressions of lncRNA in cells related to immune response, which may be involved in the pathophysiology of the diseases, including cancer and nerve, autoimmunity and eye diseases, providing significant improvements in elucidating RNA-based mechanisms in gene expression control (Yamazoe et al., 2017; Yue et al., 2018). An allele association analysis showed that the rs9517723 locus located in lncRNALoc107984558 had the strongest association, and single nucleotide polymorphism (SNP) rs9517723 was recessively associated with the risk of eye and central nervous system damage. It was also confirmed that the homozygous risk allele (TT) of lncRNA LOC107984558/rs9517723 was significantly associated with the increased expression of *UBAC2*. Gene expression analysis showed that the expression of rs9517723 TT homozygous *UBAC2* was significantly increased. Increase in *UBAC2* expression of homozygous risk allele (TT) in rs9517723 can induce overactivation of ubiquitin-related pathways, leading to eye and central nervous system lesions in BD. In the future, rs9517723 may become a useful genetic marker for the diagnosis of BD, especially in patients with central nervous system diseases (Yamazoe et al., 2017). Another study found that *lncRNA-CD244*, *lncRNA-Cox2*, and *THRIL* are expressed in immune cells, which may regulate the immune response and the production of cytokines, such as IFN-γ, TNF-α, IL-4, and IL-12, which are related to the pathogenesis of many uveitis entities (Yue et al., 2018).

At present, there are few studies on lncRNA, and research on the participation of ncRNA in BD is mainly focused on miRNA.

#### MicroRNA and Behçet’s Disease

The three main types of short ncRNA are miRNA, siRNA, and Piwi-interaction RNA; miRNA are important mediators of mammalian epigenetic gene regulation and the key regulatory factors of immune response. Mature miRNA inhibits protein synthesis and negatively regulates gene expression by recognizing the 3′UTR region of the target mRNA, degrading the target gene, or inhibiting its translation (Yao et al., 2019). A great number of studies have shown that miRNA plays a critical role in the regulation of immune response and immune cell development. A miRNA can regulate hundreds of target genes by inhibiting translation, mediating mRNA fragments, or causing RNA instability. Multiple miRNAs can cooperatively bind and regulate a single target gene (Deng et al., 2015). MiRNA regulates T-cell differentiation and plasticity by targeting its corresponding mRNAs and plays an important role in many autoimmune diseases (Lu et al., 2020).

Uncontrolled miRNA targeting is involved in signaling pathways in the pathogenesis of BD, such as TNF-α, IFN-γ, and the VEGF–VEGFR signal cascade. Downregulated miRNA targets differentially expressed genes (DEGs), associated with the adaptive immune response, including genes that play a role in T-cell and B-cell immune response, and controls several genes and transcripts associated with Th17 cells, which are involved in IFN-I response, which may indicate that BD has lost control of the two synergistic mechanisms usually associated with autoimmune response (Puccetti et al., 2018). MiR-155 is related to inflammation, effectively upregulates many immune cell lines through TLR ligands, and promotes the expression of many immune cell lines through the expression of TLR ligands and precursors. Many studies have found that miR-155 is significantly downregulated in patients with BD, which is closely related to the onset and development of BD (Karasneh et al., 2005; Alipour et al., 2017; Ahmadi et al., 2019). A study analyzed the role of miRNAs in two common uveitis: BD and Vogt-Koyanagi-Harada (VKH) syndrome, and found that in PBMCs and DCs of patients with active uveitis BD, only miR-155 expression was significantly decreased, while there was no significant difference in miRNA expression in PBMCs and DCs in patients with VKH syndrome compared with control groups. In addition, it was found that the expression of TGF-β-activated kinase 1 binding protein 2 (TAB2) increased in DCs, and luciferase reporter gene detection showed that *TAB2* is the target gene of miR-155 (Zhou et al., 2012), and downregulation of miR-155 negatively regulate the inflammatory cytokines produced by DCs (Morton et al., 2016). DCs with reduced expression of miR-155 can promote the secretion of IL-6 and IL-1 but inhibits the production of IL-10. Because DCs mainly regulate the function of T cells, the downregulated expression of miR-155 can promote the secretion of IL-17, which is negatively correlated with the production of IL-17 in allogeneic CD4^+^ T cells. Some studies have found that miR-155 regulates the Th17 immune response in patients with active BD (Na et al., 2016). The increase of Th1/Th17 ratio is usually related to BD, and Th17 cells response plays an important role in the pathogenesis of BD (Deng et al., 2015). In addition, the overexpression of miR-155 also significantly inhibits apoptosis, and the level of miR-155 is often lower in BD patients with severe clinical manifestations and higher BDCAF scores (Hatemi et al., 2019). And miR-155 targeting FoxP3 can regulate the differentiation and function of Th17 cells by inducing their differentiation into Tregs. However, miR-155 cannot effectively secrete Tregs-related cytokines, which may play a role by regulating the frequency, transcription factors, and cytokine levels of Tregs (Ahmadi et al., 2019).

In addition, studies have found that miRNA, which controls members of the TLRs and JAK/STAT pathways, is downregulated. These two molecular signals are involved in autoimmune diseases, and are also active in BD (Puccetti et al., 2018). Some studies have found that the miR-146a variant rs2910164 is closely associated with BD in Chinese population (Deng et al., 2015). The protein levels of mature miR-146a transcripts and IL-17, TNF-α, and IL-1β in the PBMCs of BD patients with rs2910164 CC genotype are lower than those of GG genotype (Coit et al., 2018), which is more common in BD patients. The CC genotype of rs2910164 has a protective effect on BD. In BD patients, miR-146a is a negative regulator of innate immunity (Morton et al., 2016), and is also a key regulator of IFN-I pathway (Yokota et al., 1992). It is highly expressed in Tregs, which is considered necessary for the inhibitory function of Tregs, and affects the ability of these cells to inhibit Th1 cell’s response through STAT1. STAT1 is an important transcriptional factor in the differentiation of Th1 cells. MiR-146a can inhibit the transition of Tregs to Th1-like cells (Ahmadi et al., 2019). A previous study confirmed that miR-146a plays a negative feedback regulation role in TLR signals by targeting TRAF6 and IL-1R related kinase (IRAK) (Taganov et al., 2006), which downregulation may play an important role in the occurrence of BD. In the miRNA study of BD, the regulation of Th17 cellular activity has become a popular topic. MiR-23b can reduce the production of IFN-γ and IL-17 by reducing the expression of Notch pathway genes (Coit et al., 2018). The expression of miR-23b in CD4^+^ T cells of active BD patients was significantly lower than that of normal controls or inactive BD patients, and the active forms of Notch target genes Hes-1 and Notch1 were significantly increased in PBMCs and CD4^+^ T cells. Reduced miR-23b expression may contribute to the activation of Notch pathway and the increase of Th1/Th17 cells in BD patients. Because STAT3 plays a key role in the differentiation of Th17 lymphocytes, the increased activation of Notch pathway associated with STAT3 phosphorylation may promote Th17 cell response in patients with active BD (Qi et al., 2014). Studies have also confirmed that the level of STAT3 phosphorylation is increased in patients with BD. Therefore, the regulation of Notch pathway may provide a novel treatment for BD. In addition, miR-23b is one of the miRNA involved in the differentiation of Tregs. The low expression of miR-23b is consistent with the finding of decreased Tregs levels in BD patients in early studies (Nanke et al., 2008), which was confirmed in another study (Morton et al., 2016).

SNPs may change the properties of miRNA by changing the expression or maturation of miRNA. Therefore, SNP in the process of mature miRNA may be related to autoimmune or autoinflammatory diseases. Researchers found that the frequencies of miR-196a2/rs11614913 TT genotype and T allele in BD patients with arthritis were significantly higher than those in non-arthritis BD patients. The expression of miR-196a is decreased in individuals with rs11614913 TT genotype, while the expression of Bach1 is increased. Luciferase report experiment confirmed that Bach1 was the target gene of miR-196a, and there was a negative correlation between miR-196a and Bach1 (Qi et al., 2013). Bach1 is a mammalian transcriptional inhibitor of heme oxygenase-1 (HO-1) (Sun et al., 2002). The expression of HO-1 in the PBMCs of patients with BD is decreased. Bach1/HO-1 is a well-known oxidative stress signal pathway and participates in the pathogenesis of several inflammatory diseases. Rs11614913 may lead to the imbalance of Bach1/HO-1 pathway through the change of miR-196a expression. It affects the expression of pro-inflammatory cytokines and leads to the pathogenesis of BD. In addition, researchers used quantitative PCR to detect the expression levels of four selected miRNAs (miR-638, miR-4488, miR-3591-3p, and miR-1915), and to explore their relationship with TNF-α and IL-6 production. It was found that the expression of miR-638 and miR-4488 in the PBMCs of patients with stable BD was significantly lower than that of healthy controls. Stimulation of LPS can increase the level of miR-4488 in the PBMCs of patients with stable BD to the level of healthy controls. By contrast, the expression of miR-3591-3p in PBMC of active BD patients was significantly higher than that of BD patients in remission stage. Transfection of miR-3591-3p mimic could increase the IL-6 mRNA level of THP-1 cells stimulated by LPS, but the specific mechanism has not been elucidated (Woo et al., 2016). In other studies, it has also been found that some of the upregulation of miRNA is also related to BD.

Compared with the control group, miR-25, miR-106b, miR-326, and miR-93 in peripheral blood of BD patients were significantly up-regulated. MiR-25, miR-106b, and miR-93 are located in the miR-106b-25 cluster and participate in the regulation of TGF-β pathway (Ahmadi et al., 2019). TGF-β plays an important role in the development of Tregs by inducing FoxP3. Therefore, the increased expression of miR-106b-25 associated with Tregs may disrupt the signal pathway of TGF-β and affect the differentiation of Tregs (De Santis et al., 2010). MiR-326 regulates the differentiation of Th17 cells by targeting Ets-1, a member of the ETS transcription factor family. The proportion of Th17 cells in BD patients is significantly increased, accompanied by increased gene expression levels of IL-17, IL-23, and retinoic acid-related orphan receptor. The balance of Th17/Tregs is broken, which may play a role in the occurrence and development of BD. The current evidence suggests that the impairment of inflammatory regulation in BD patients may be mediated by abnormal T-cell homeostasis. Genetic variations in the miRNA gene associated with BD have been shown to promote the phenotypic transformation of a disease, increase the production of pro-inflammatory cytokines, and reduce the expansion of anti-inflammatory Tregs. A study analyzed the association of miR-182/rs76481776 in 420 BD and 1200 controls, which confirmed that miR-182/rs76481776 is related to BD. MiR-182 can target the 3′UTR of *FoxO1*, resulting in the degradation of FoxO1, and FoxO1 controls the development and function of Tregs. Therefore, Tregs decrease and assist T cell clone proliferation. At the same time, miR-182 is IL-2-induced miRNA, which regulates the specialization and stability of Tregs, and is the key switch for Tregs differentiation. Treg is a subgroup of T cells, which regulates the immune system and maintains tolerance to autoantigens, thus controlling the occurrence of dominant autoimmune diseases (Yu et al., 2014).

## The Interaction Between *TLR4* and Gut Microbiota May Be Associated with the Development of Behçet’s Disease

As mentioned earlier, *TLR4* showed hypomethylation and increased expression in Iranian BD patients (Horie et al., 2009; Kolahi et al., 2020). In addition, the researchers detected and analyzed the association between nine SNPs in *TLR4* and BD through direct sequencing, and found that the *TLR4* polymorphism may increase the risk of BD in the Japanese population (Meguro et al., 2008), which has also been confirmed in the Korean and Italian populations (Meguro et al., 2008; Boiardi et al., 2009). The expression of *TLR4* was also increased in intestinal lesions of BD patients, which may be related to intestinal abnormalities in BD patients (Nara et al., 2008). Interestingly, another study found that systemic exposure to TLR ligands caused rapid α (1,2)-fucosylation of small intestine epithelial cells (IECs) in mice, which needed the sensing of TLR agonists, the IL-23 production by DCs, activation of innate lymphoid cells and expression of fucosyltransferase 2 (FUT2) by IL-22-stimulated IECs (Pickard et al., 2014; Xavier et al., 2015). It can be seen from the above that the TLR4-IL-23-IL-22-FUT2 pathway may be a potential pathway for the interaction between genetics and GM to participate in the pathogenesis of BD, but the specific mechanism needs further studies.

## Conclusion

**FIGURE 1:**
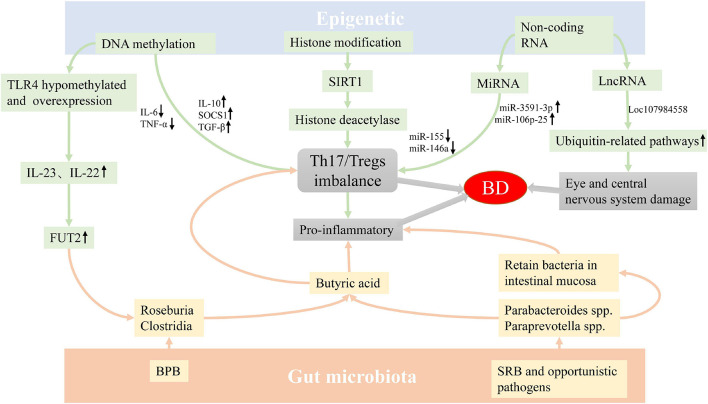
The possible mechanism of epigenetics factors and GM involving the pathogenesis of BD. The imbalance of GM and epigenetics abnormality may be involved in the occurrence of BD by regulating the differentiation of T cells. In addition, the abnormality of conditional pathogenic bacteria may cause inflammation by retaining bacteria in intestinal mucosa, and the abnormality of LncRNA can also cause eye and central nervous system damage in patients with BD. Abbreviation: GM: Gut microbiota, BD: Behçet’s disease, BPB: butyrate-producing bacteria, SRB: sulfate-reducing bacteria.

In summary, the abnormalities of GM and epigenetic factors may provide a susceptible background for the occurrence of BD through a variety of mechanisms (Figure 1). However, there are few related studies at present, and whether GM and genetic components affect the development of BD has not yet been clearly elucidated, and the specific mechanism of epigenetics on BD is also unclear. The future direction of study may be to combine environmental factors with genetic factors to explore whether they interact with each other and participate in the occurrence of diseases, so as to reveal the occurrence and development of BD as comprehensively as possible, thereby promoting the understanding of diseases, and providing new targets and approaches for disease prevention, diagnosis and treatment.

## Author Contributions

HY and XM conceptualized and wrote this manuscript. XW, GZ, GT, WW, FZ, and DT had critically reviewed the manuscript. All authors contributed to the article and approved the submitted version.

## Conflict of Interest

The authors declare that the research was conducted in the absence of any commercial or financial relationships that could be construed as a potential conflict of interest.

## Publisher’s Note

All claims expressed in this article are solely those of the authors and do not necessarily represent those of their affiliated organizations, or those of the publisher, the editors and the reviewers. Any product that may be evaluated in this article, or claim that may be made by its manufacturer, is not guaranteed or endorsed by the publisher.

## References

[B1] Abdi A. Khabazi A. Sakhinia E. Alipour S. Talei M. Babaloo Z. (2018). Evaluation of SOCS1 methylation in patients with Behçet’s disease. *Immunol. Lett.* 203 15–20. 10.1016/j.imlet.2018.07.001 29990515

[B2] Acosta-Rodriguez E. V. Napolitani G. Lanzavecchia A. Sallusto F. (2007). Interleukins 1β and 6 but not transforming growth factor-β are essential for the differentiation of interleukin 17–producing human T helper cells. *Nat. Immunol.* 8 942–949. 10.1038/ni1496 17676045

[B3] Ahmadi M. Yousefi M. Abbaspour-Aghdam S. Dolati S. Aghebati-Maleki L. Eghbal-Fard S. (2019). Disturbed Th17/Treg balance, cytokines, and miRNAs in peripheral blood of patients with Behçet’s disease. *J. Cell. Physiol.* 234 3985–3994. 10.1002/jcp.27207 30317557

[B4] Akbaba T. H. Sag E. Balci-Peynircioglu B. Ozen S. (2020). Epigenetics for Clinicians from the Perspective of Pediatric Rheumatic Diseases. *Curr. Rheumatol. Rep.* 22:46. 10.1007/s11926-020-00912-9 32654092

[B5] Alipour S. Nouri M. Khabbazi A. Samadi N. Babaloo Z. Abolhasani S. (2018). Hypermethylation of IL-10 gene is responsible for its low mRNA expression in Behçet’s disease. *J. Cell. Biochem.* 119 6614–6622. 10.1002/jcb.26809 29719061

[B6] Alipour S. Nouri M. Sakhinia E. Samadi N. Roshanravan N. Ghavami A. (2017). Epigenetic alterations in chronic disease focusing on Behçet’s disease: review. *Biomed. Pharmacother.* 91 526–533. 28482290 10.1016/j.biopha.2017.04.106

[B7] Alipour S. Sakhinia E. Khabbazi A. Samadi N. Babaloo Z. Azad M. (2020). Methylation Status of Interleukin-6 Gene Promoter in Patients with Behçet’s Disease. *Reumatol*. *Clin.* 16 229–234. 10.1016/j.reuma.2018.06.006 30076035

[B8] Allis C. D. Jenuwein T. (2016). The molecular hallmarks of epigenetic control. *Nat. Rev. Genet.* 17 487–500. 10.1038/nrg.2016.59 27346641

[B9] Angeloni A. Bogdanovic O. (2019). Enhancer DNA methylation: implications for gene regulation. *Essays Biochem.* 63 707–715. 10.1042/EBC20190030 31551326

[B10] Arpaia N. Campbell C. Fan X. Dikiy S. van der Veeken J. deRoos P. (2013). Metabolites produced by commensal bacteria promote peripheral regulatory T-cell generation. *Nature* 504 451–455. 10.1038/nature12726 24226773 PMC3869884

[B11] Asadi S. Khabbazi A. Alipour S. Abolhasani S. Haji J. Amjadi H. (2020). Promoter methylation of Bax and Bcl2 genes and their expression in patients with Behçet’s disease. *Int. J. Immunogenet.* 47 309–317. 10.1111/iji.12473 31916399

[B12] Aziz S. G. Aziz S. G. Khabbazi A. Alipour S. (2020). The methylation status of TNF-α and SOCS3 promoters and the regulation of these gene expressions in patients with Behçet’s disease. *Biomarkers* 25 384–390. 32475174 10.1080/1354750X.2020.1754912

[B13] Bannister A. J. Kouzarides T. (2011). Regulation of chromatin by histone modifications. *Cell. Res.* 21 381–395. 10.1038/cr.2011.22 21321607 PMC3193420

[B14] Barrere-Cain R. Allard P. (2020). An Understudied Dimension:Why Age Needs to Be Considered When Studying Epigenetic-Environment Interactions. *Epigenet. Insights* 13:2516865720947014. 10.1177/2516865720947014 32864568 PMC7430070

[B15] Bartlett D. B. Willis L. H. Slentz C. A. Hoselton A. Kelly L. Huebner J. L. (2018). Ten weeks of high-intensity interval walk training is associated with reduced disease activity and improved innate immune function in older adults with rheumatoid arthritis: a pilot study. *Arthritis Res. Ther.* 20:127. 10.1186/s13075-018-1624-x 29898765 PMC6001166

[B16] Beier U. H. Wang L. Bhatti T. R. Liu Y. Han R. Ge G. (2011). Sirtuin-1 targeting promotes Foxp3+ T-regulatory cell function and prolongs allograft survival. *Mol. Cell. Biol.* 31 1022–1029. 10.1128/MCB.01206-10 21199917 PMC3067815

[B17] Boiardi L. Atzeni F. Casali B. Farnetti E. Nicoli D. Pipitone N. (2009). Toll-like receptor 4 (TLR4) gene polymorphisms in Italian patients with Behçet’s disease. *Clin. Exp. Rheumatol*. 27 S43–S47. 19796532

[B18] Brandl C. Bucci L. Schett G. Zaiss M. M. (2021). Crossing the barriers: revisiting the gut feeling in rheumatoid arthritis. *Eur. J. Immunol.* 51 798–810. 10.1002/eji.202048876 33594693

[B19] Chadha S. Wang L. Hancock W. W. Beier U. H. (2019). ). Sirtuin−1 in immunotherapy: a Janus−headed target. *J. Leukoc. Biol.* 106 337–343. 30605226 10.1002/JLB.2RU1118-422R PMC7477756

[B20] Chen J. Zhou Y. Mueller-Steiner S. Chen L. F. Kwon H. Yi S. (2005). SIRT1 protects against microglia-dependent amyloid-beta toxicity through inhibiting NF-kappaB signaling. *J. Biol. Chem.* 280 40364–40374. 10.1074/jbc.M509329200 16183991

[B21] Chen S. C. Chuang C. T. Chu M. Y. Sheu S. J. (2017). Patterns and Etiologies of Uveitis at a Tertiary Referral Center in Taiwan. *Ocul. Immunol. Inflamm.* 25 S31–S38. 10.1080/09273948.2016.1189577 27463023

[B22] Cho S. B. Cho S. Bang D. (2012). New insights in the clinical understanding of Behçet’s disease. *Yonsei Med. J.* 53 35–42. 10.3349/ymj.2012.53.1.35 22187230 PMC3250322

[B23] Coit P. Direskeneli H. Sawalha A. H. (2018). An update on the role of epigenetics in systemic vasculitis. *Curr. Opin. Rheumatol.* 30 4–15. 28957963 10.1097/BOR.0000000000000451 PMC5805392

[B24] Consolandi C. Turroni S. Emmi G. Severgnini M. Fiori J. Peano C. (2015). Behçet’s syndrome patients exhibit specific microbiome signature. *Autoimmun. Rev.* 14 269–276. 10.1016/j.autrev.2014.11.009 25435420

[B25] De Santis G. Ferracin M. Biondani A. Caniatti L. Rosaria Tola M. Castellazzi M. (2010). Altered miRNA expression in T regulatory cells in course of multiple sclerosis. *J. Neuroimmunol.* 226 165–171. 20637509 10.1016/j.jneuroim.2010.06.009

[B26] Deng X. Su Y. Wu H. Wu R. Zhang P. Dai Y. (2015). The Role of MicroRNAs in Autoimmune Diseases with Skin Involvement. *Scand. J. Immunol.* 81 153–165. 10.1111/sji.12261 25430682

[B27] Ehrlich M. Gama-Sosa M. A. Huang L. H. Midgett R. M. Kuo K. C. McCune R. A. (1982). Amount and distribution of 5-methylcytosine in human DNA from different types of tissues of cells. *Nucleic Acids Res*. 10 2709–2721. 10.1093/nar/10.8.2709 7079182 PMC320645

[B28] Gardner P. J. Joshi L. Lee R. W. J. Dick A. D. Adamson P. Calder V. L. (2013). SIRT1 activation protects against autoimmune T cell-driven retinal disease in mice via inhibition of IL-2/Stat5 signaling. *J. Autoimmun.* 42 117–129. 23395551 10.1016/j.jaut.2013.01.011

[B29] Guenane H. Hartani D. Chachoua L. Lahlou-Boukoffa O. S. Mazari F. Touil-Boukoffa C. (2006). Production des cytokines Th1/Th2 et du monoxyde d’azote au cours de l’uvéite "Behçet" et de l’uvéite "idiopathique" [Production of Th1/Th2 cytokines and nitric oxide in Behçet’s uveitis and idiopathic uveitis]. *J. Fr. Ophtalmol.* 29 146–152. 10.1016/s0181-5512(06)73762-7 16523155

[B30] Hamzaoui K. Bouali E. Ghorbel I. Khanfir M. Houman H. Hamzaoui A. (2011). Expression of Th-17 and RORγt mRNA in Behçet’s Disease. *Med. Sci. Monit.* 17 CR227–CR234. 10.12659/msm.881720 21455110 PMC3539514

[B31] Han X. Wang Y. Zhang P. Zhu M. Li L. Mao X. (2021). Kazak faecal microbiota transplantation induces short-chain fatty acids that promote glucagon-like peptide-1 secretion by regulating gut microbiota in db/db mice. *Pharm. Biol*. 59 1077–1087. 10.1080/13880209.2021.1954667 34392792 PMC8366640

[B32] Hanson M. A. Gluckman P. D. (2008). Developmental origins of health and disease: new insights. *Basic. Clin. Pharmacol. Toxicol.* 102 90–93. 10.1111/j.1742-7843.2007.00186.x 18226060

[B33] Hatemi G. Seyahi E. Fresko I. Talarico R. Hamuryudan V. (2019). One year in review 2019: behçet’s syndrome. *Clin. Exp. Rheumatol.* 37 3–17. 31856939

[B34] He J. Chan T. Hong X. Zheng F. Zhu C. Yin L. (2020). Microbiome and Metabolome Analyses Reveal the Disruption of Lipid Metabolism in Systemic Lupus Erythematosus. *Front. Immunol.* 11:1703. 10.3389/fimmu.2020.01703 32849599 PMC7411142

[B35] Horie Y. Meguro A. Ota M. Kitaichi N. Katsuyama Y. Takemoto Y. (2009). Association of TLR4 polymorphisms with Behcet’s disease in a Korean population. *Rheumatology* 48 638–642. 19395541 10.1093/rheumatology/kep077

[B36] Hou S. Li N. Liao X. Kijlstra A. Yang P. (2020). Uveitis genetics. *Exp. Eye Res.* 190:107853. 10.1016/j.exer.2019.107853 31669406

[B37] Hou S. Shu Q. Jiang Z. Chen Y. Li F. Chen F. (2012). Replication study confirms the association between UBAC2 and Behçet’s disease in two independent Chinese sets of patients and controls. *Arthritis Res. Ther.* 14:R70. 10.1186/ar3789 22455605 PMC3446441

[B38] Hou S. Yang P. Du L. Zhou H. Lin X. Liu X. (2008). SUMO4 gene polymorphisms in Chinese Han patients with Behçet’s disease. *Clin. Immunol.* 129 170–175. 10.1016/j.clim.2008.06.006 18657476

[B39] Jun J. B. Kim H. J. Kazmi S. Z. Kang T. Kim K. B. Kang M. J. (2020). Significant Decline in the Incidence of Behcet’s Disease in South Korea: a Nationwide Population-Based Study (2004-2017). *Arthritis Care Res.* 10.1002/acr.24408 [Epub Online ahead of print]. 32770715

[B40] Kaabachi W. Bouali E. Berraïes A. Dhifallh I. B. Hamdi B. Hamzaoui K. (2017). Interleukin-26 is overexpressed in Behçet’s disease and enhances Th17 related -cytokines. *Immunol. Lett*. 190 177–184. 10.1016/j.imlet.2017.08.008 28811236

[B41] Kaabachi W. Khaouthar M. Hamdi B. Khalfallah I. Ammar J. Hamzaoui K. (2019). Th 9 cells in Behçet disease: possible involvement of IL-9 in pulmonary manifestations. *Immunol. Lett*. 211 3–12. 10.1016/j.imlet.2019.05.004 31075294

[B42] Kamoun M. Ben Dhifallah I. Karray E. Zakraoui L. Hamzaoui K. (2010). Association of small ubiquitin-like modifier 4 (SUMO4) polymorphisms in a Tunisian population with Behçet’s disease. *Clin. Exp. Rheumatol*. 28 S45–S49. 20868570

[B43] Karasneh J. Gül A. Ollier W. E. Silman A. J. Worthington J. (2005). Whole-genome screening for susceptibility genes in multicase families with Behçet’s disease. *Arthritis Rheumatol.* 52 1836–1842. 10.1002/art.21060 15934084

[B44] Kolahi S. Rashtchizadeh N. Mahdavi A. M. Farhadi J. Khabbazi A. Sakhinia E. (2020). Evaluation of DNA methylation status of toll−like receptors 2 and 4 promoters in Behcet’s disease. *J. Gene Med.* 22:e3234. 10.1002/jgm.3234 32449979

[B45] Kubota S. Kurihara T. Mochimaru H. Satofuka S. Noda K. Ozawa Y. (2009). Prevention of ocular inflammation in endotoxin-induced uveitis with resveratrol by inhibiting oxidative damage andnuclear factor-kappaB activation. *Invest. Ophthalmol. Vis. Sci*. 50 3512–3519. 10.1167/iovs.08-2666 19279313

[B46] Leccese P. Alpsoy E. (2019). Behçet’s Disease: an Overview of Etiopathogenesis. *Front. Immunol.* 10:1067. 10.3389/fimmu.2019.01067 31134098 PMC6523006

[B47] Liang L. Zhou Q. Feng L. (2021). Decreased microRNA-155 in Behcet’s disease leads to defective control of autophagy thereby stimulating excessive proinflammatory cytokine production. *Arthritis Res. Ther*. 23:135. 10.1186/s13075-021-02517-8 33957967 PMC8101176

[B48] Lopalco G. Lucherini O. M. Lopalco A. Venerito V. Fabiani C. Frediani B. (2017). Cytokine Signatures in Mucocutaneous and Ocular Behçet’s Disease. *Front. Immunol*. 8:200. 10.3389/fimmu.2017.00200 28289419 PMC5327443

[B49] Lu S. Yadav A. K. Qiao X. (2020). Identification of potential miRNA-mRNA interaction network in bone marrow T cells of acquired aplastic anemia. *Hematology* 25 168–175. 10.1080/16078454.2020.1757332 32338587

[B50] Maldini C. Druce K. Basu N. LaValley M. P. Mahr A. (2018). Exploring the variability in Behçet’s disease prevalence: a meta-analytical approach. *Rheumatology* 57 185–195. 10.1093/rheumatology/kew486 28339670

[B51] Meguro A. Ota M. Katsuyama Y. Oka A. Ohno S. Inoko H. (2008). Association of the toll-like receptor 4 gene polymorphisms with Behcet’s disease. *Ann. Rheum. Dis*. 67 725–727. 10.1136/ard.2007.079871 18408113

[B52] Morton L. T. Situnayake D. Wallace G. R. (2016). Genetics of Behçet’s disease. *Curr. Opin. Rheumatol.* 28 39–44. 10.1097/BOR.0000000000000234 26599381

[B53] Muhammad J. S. Ishaq M. Ahmed K. (2019). Genetics and Epigenetics Mechanism in the Pathogenesis of Behçet’s Disease. *Curr. Rheumatol. Rev.* 15 7–13. 10.2174/1573397114666180521090335 29779484

[B54] Na S. Y. Park M. J. Park S. Lee E. S. (2016). MicroRNA-155 regulates the Th17 immune response by targeting Ets-1 in Behçet’s disease. *Clin. Exp. Rheumatol.* 34 S56–S63. 27156371

[B55] Nakamura J. Meguro A. Ishii G. Mihara T. Takeuchi M. Mizuki Y. (2019). The association analysis between HLA-A*26 and Behçet’s disease. *Sci. Rep.* 9:4426. 10.1038/s41598-019-40824-y 30872678 PMC6418292

[B56] Nanke Y. Kotake S. Goto M. Ujihara H. Matsubara M. Kamatani N. (2008). Decreased percentages of regulatory T cells in peripheral blood of patients with Behçet’s disease before ocular attack: a possible predictive marker of ocular attack. *Mod. Rheumatol.* 18 354–358. 10.1007/s10165-008-0064-x 18427720

[B57] Nara K. Kurokawa M. S. Chiba S. Yoshikawa H. Tsukikawa S. Matsuda T. (2008). Involvement of innate immunity in the pathogenesis of intestinal Behçet’s disease. *Clin. Exp. Immunol.* 152 245–251. 18336589 10.1111/j.1365-2249.2008.03626.x PMC2384098

[B58] Oezguen N. Yalcinkaya N. Kücükali C. I. Dahdouli M. Hollister E. B. Luna R. A. (2019). Microbiota stratification identifies disease-specific alterations in neuro-Behçet’s disease and multiple sclerosis. *Clin. Exp. Rheumatol.* 37 58–66. 31172918

[B59] Ortiz-Fernández L. Sawalha A. H. (2021). Genetics of Behçet’s Disease: functional Genetic Analysis and Estimating Disease Heritability. *Front. Med.* 8:625710. 10.3389/fmed.2021.625710 33644100 PMC7907152

[B60] Pickard J. M. Maurice C. F. Kinnebrew M. A. Abt M. C. Schenten D. Golovkina T. V. (2014). Rapid fucosylation of intestinal epithelium sustains host-commensal symbiosis in sickness. *Nature* 514 638–641. 10.1038/nature13823 25274297 PMC4214913

[B61] Puccetti A. Pelosi A. Fiore P. F. Patuzzo G. Lunardi C. Dolcino M. (2018). MicroRNA Expression Profiling in Behçet’s Disease. *J. Immunol. Res.* 2018:2405150. 10.1155/2018/2405150 29854829 PMC5964440

[B62] Qi J. Hou S. Zhang Q. Liao D. Wei L. Fang J. (2013). A functional variant of pre-miRNA-196a2 confers risk for Behcet’s disease but not for Vogt-Koyanagi-Harada syndrome or AAU in ankylosing spondylitis. *Hum. Genet.* 132 1395–1404. 10.1007/s00439-013-1346-8 23928854

[B63] Qi J. Yang Y. Hou S. Qiao Y. Wang Q. Yu H. (2014). Increased Notch pathway activation in Behçet’s disease. *Rheumatology* 53 810–820. 10.1093/rheumatology/ket438 24446471

[B64] Renauer P. Coit P. Sawalha A. H. (2016). Epigenetics and Vasculitis: a Comprehensive Review. *Clin. Rev. Allergy Immunol.* 50 357–366. 10.1007/s12016-015-8495-6 26093659

[B65] Round J. L. Mazmanian S. K. (2009). The gut microbiota shapes intestinal immune responses during health and disease. *Nat. Rev. Immunol.* 9 313–323. 10.1038/nri2515 19343057 PMC4095778

[B66] Shimizu J. Kubota T. Takada E. Takai K. Fujiwara N. Arimitsu N. (2016). Bifidobacteria Abundance-Featured Gut Microbiota Compositional Change in Patients with Behçet’s disease. *PLoS One* 11:e0153746. 10.1371/journal.pone.0153746 27105322 PMC4841557

[B67] Shimizu J. Kubota T. Takada E. Takai K. Fujiwara N. Arimitsu N. (2019). Relative abundance of *Megamonas hypermegale* and *Butyrivibrio* species decreased in the intestine and its possible association with the T cell aberration by metabolite alteration in patients with Behçet’s disease (210 characters). *Clin. Rheumatol.* 38 1437–1445. 10.1007/s10067-018-04419-8 30628011

[B68] Silva H. G. Sobral R. S. Magalhães A. P. Morais-Cecílio L. Costa M. M. R. (2020). Genome-Wide Identification of Epigenetic Regulators in *Quercus suber* L. *Int. J. Mol. Sci.* 21:3783. 10.3390/ijms21113783 32471127 PMC7313042

[B69] Sun J. Hoshino H. Takaku K. Nakajima O. Muto A. Suzuki H. (2002). Hemoprotein Bach1 regulates enhancer availability of heme oxygenase-1 gene. *EMBO J*. 21 5216–5224. 10.1093/emboj/cdf516 12356737 PMC129038

[B70] Taganov K. D. Boldin M. P. Chang K. J. Baltimore D. (2006). NF-kappaB-dependent induction of microRNA miR-146, an inhibitor targeted to signaling proteins of innate immune responses. *Proc. Natl. Acad. Sci. U. S. A.* 103 12481–12486. 10.1073/pnas.0605298103 16885212 PMC1567904

[B71] Takeuchi M. Mizuki N. Meguro A. Ombrello M. J. Kirino Y. Satorius C. (2017). Dense genotyping of immune-related loci implicates host responses to microbial exposure in Behçet’s disease susceptibility. *Nat. Genet.* 49 438–443. 10.1038/ng.3786 28166214 PMC5369770

[B72] Tecer D. Gogus F. Kalkanci A. Erdogan M. Hasanreisoglu M. Ergin Ç (2020). Succinivibrionaceae is dominant family in fecal microbiota of Behçet’s Syndrome patients with uveitis. *PLoS One* 15:e0241691. 10.1371/journal.pone.0241691 33125440 PMC7598488

[B73] Todaro M. Zerilli M. Triolo G. Iovino F. Patti M. Accardo-Palumbo A. (2005). NF-κB protects Behçet’s disease T cells against CD95-induced apoptosis up-regulating antiapoptotic proteins. *Arthritis Rheum.* 52 2179–2191. 10.1002/art.21145 15986355

[B74] van der Houwen T. B. van Laar J. A. M. Kappen J. H. van Hagen P. M. de Zoete M. R. van Muijlwijk G. H. (2020). Behçet’s Disease Under Microbiotic Surveillance? A Combined Analysis of Two Cohorts of Behçet’s Disease Patients. *Front. Immunol.* 11:1192. 10.3389/fimmu.2020.01192 32595645 PMC7303268

[B75] Vural S. Kerl K. Doǧan P. E. Vollmer S. Puchta U. He M. (2021). Lesional activation of Tc 17 cells in Behçet’s disease and psoriasis supports HLA-class I-mediated autoimmune responses. *Br. J. Dermatol.* 10.1111/bjd.20643 [Epub online ahead of print]. 34254298

[B76] Wilson N. J. Boniface K. Chan J. R. McKenzie B. S. Blumenschein W. M. Mattson J. D. (2007). Development, cytokine profile and function of human interleukin 17–producing helper T cells. *Nat. Immunol.* 8 950–957. 10.1038/ni1497 17676044

[B77] Woo M. Y. Yun S. J. Cho O. Kim K. Lee E. S. Park S. (2016). MicroRNAs differentially expressed in Behçet disease are involved in interleukin-6 production. *J. Inflamm.* 13:22. 10.1186/s12950-016-0130-7 27441030 PMC4952146

[B78] Xavier J. M. Shahram F. Sousa I. Davatchi F. Matos M. Abdollahi B. S. (2015). FUT2: filling the gap between genes and environment in Behçet’s disease? *Ann. Rheum. Dis*. 74 618–624. 10.1136/annrheumdis-2013-204475 24326010

[B79] Yamazoe K. Meguro A. Takeuchi M. Shibuya E. Ohno S. Mizuki N. (2017). Comprehensive analysis of the association between UBAC2 polymorphisms and Behçet’s disease in a Japanese population. *Sci. Rep.* 7:742. 10.1038/s41598-017-00877-3 PMC5429716 28389674

[B80] Yan J. B. Luo M. M. Chen Z. Y. He B. H. (2020). The Function and Role of the Th17/Treg Cell Balance in Inflammatory Bowel Disease. *J. Immunol. Res.* 2020:8813558. 10.1155/2020/8813558 33381606 PMC7755495

[B81] Yang P. Zhong Z. Du L. Li F. Chen Z. Zhu Y. (2021). Prevalence and clinical features of systemic diseases in Chinese patients with uveitis. *Br. J. Ophthalmol.* 105 75–82. 10.1136/bjophthalmol-2020-315960 32188681

[B82] Yao Q. Chen Y. Zhou X. (2019). The roles of microRNAs in epigenetic regulation. *Curr. Opin. Chem. Biol.* 51 11–17. 10.1016/j.cbpa.2019.01.024 30825741

[B83] Ye Z. Zhang N. Wu C. Zhang X. Wang Q. Huang X. (2018). A metagenomic study of the gut microbiome in Behçet’s disease. *Microbiome* 6:135. 10.1186/s40168-018-0520-6 30077182 PMC6091101

[B84] Yeung F. Hoberg J. E. Ramsey C. S. Keller M. D. Jones D. R. Frye R. A. (2004). Modulation of NF-kappaB-dependent transcription and cell survival by the SIRT1 deacetylase. *EMBO J.* 23 2369–2380. 10.1038/sj.emboj.7600244 15152190 PMC423286

[B85] Yokota K. Hayashi S. Fujii N. Yoshikawa K. Kotake S. Isogai E. (1992). Antibody response to oral streptococci in Behçet’s disease. *Microbiol. Immunol.* 36 815–822. 1474932 10.1111/j.1348-0421.1992.tb02083.x

[B86] Yu H. Du L. Yi S. Wang Q. Zhu Y. Qiu Y. (2019). Epigenome-wide association study identifies Behçet’s disease-associated methylation loci in Han Chinese. *Rheumatology* 58 1574–1584. 10.1093/rheumatology/kez043 30863869

[B87] Yu H. Liu Y. Bai L. Kijlstra A. Yang P. (2014). Predisposition to Behçet’s disease and VKH syndrome by genetic variants of miR-182. *J. Mol. Med.* 92 961–967. 10.1007/s00109-014-1159-9 24801147

[B88] Yue Y. Zhang J. Yang L. Liu S. Qi J. Cao Q. (2018). Association of Long Noncoding RNAs Polymorphisms With Ankylosing Spondylitis, Vogt-Koyanagi-Harada Disease, and Behçet’s disease. *Invest. Ophthalmol. Vis. Sci.* 59 1158–1166. 10.1167/iovs.17-23247 29490353

[B89] Zhang X. Shi L. Sun T. Guo K. Geng S. (2021). Dysbiosis of gut microbiota and its correlation with dysregulation of cytokines in psoriasis patients. *BMC Microbiol.* 21:78. 10.1186/s12866-021-02125-1 33685393 PMC7941898

[B90] Zhou Q. Xiao X. Wang C. Zhang X. Li F. Zhou Y. (2012). Decreased microRNA-155 expression in ocular Behçet’s disease but not in Vogt Koyanagi Harada syndrome. *Invest. Ophthalmol. Vis. Sci.* 53 5665–5674. 10.1167/iovs.12-9832 22815348

[B91] Zhu Y. Qiu Y. Yu H. Yi S. Su W. Kijlstra A. (2017). Aberrant DNA methylation of GATA binding protein 3 (GATA3), interleukin-4 (IL-4), and transforming growth factor-β (TGF-β) promoters in Behçet’s disease. *Oncotarget* 8 64263–64272. 10.18632/oncotarget.19500 28969068 PMC5610000

